# P-592. Rapid initiation of antiretroviral therapy under the Treat-All policy reduces loss to follow-up and virological failure in routine HIV care settings in China: A retrospective cohort study (2016-2022)

**DOI:** 10.1093/ofid/ofae631.790

**Published:** 2025-01-29

**Authors:** Huan Xia, Ping Ma

**Affiliations:** Tianjin Second People's Hospital, Tianjin, Tianjin, China; Tianjin Second People's Hospital, Tianjin, Tianjin, China

## Abstract

**Background:**

Following the World Health Organization's guidelines for rapid antiretroviral therapy (ART) initiation [≤ 7 days after human immunodeficiency virus (HIV) diagnosis], China implemented Treat-All in 2016 and has made significant efforts to provide timely ART since 2017. The purpose of this study was to evaluate the effects of rapid ART on loss to follow-up (LTFU) and virological failure compared to those of delayed ART.

Trends in rapid ART initiation (A) and time from HIV diagnosis to treatment initiation (B) from June 2016 to December 2022.

**Figure:**
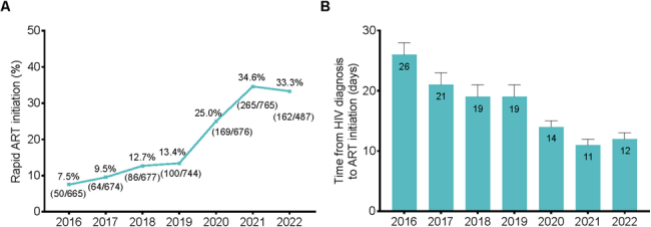

**Methods:**

This research included newly diagnosed HIV-infected adults from Tianjin, China between June 2016 and December 2022. Our primary outcome was LTFU (more than 90 days after the prior drug pick-up or clinical visit) at 12 months following enrollment. The secondary outcome was 12-month virological failure after treatment initiation. Furthermore, Kaplan-Meier estimators were utilized to examine the LTFU time for rapid and delayed ART initiation. Moreover, the correlation between rapid ART initiation and LTFU was evaluated using univariate and multivariable Cox regression, whereas the relationship between rapid ART and 12-month virological failure was investigated *via* logistic regression.

Kaplan-Meier survival curve of loss to follow-up in ART initiation group.

**Figure:**
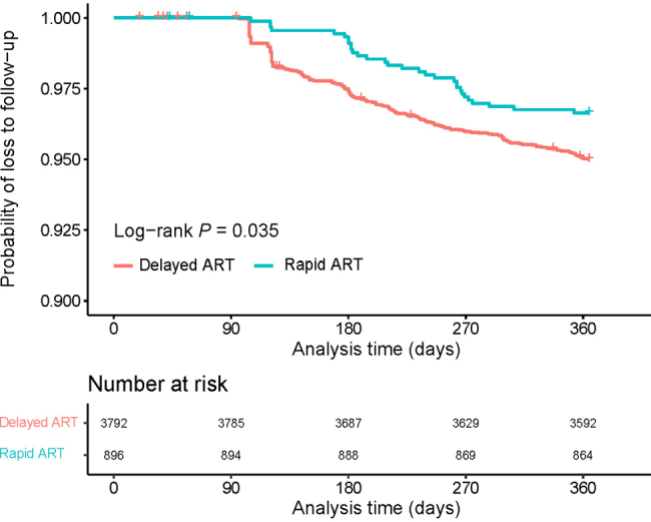

**Results:**

A total of 896 (19.1%) of 4688 participants received ART ≤ 7 days post-HIV diagnosis. The rate of rapid ART initiation has increased from 7.5% in 2016 to 33.3% by 2022. Rapid ART was less common in persons with baseline CD4 counts of 350 - 499 and ≥ 500 compared to those < 200. Furthermore, it was also less prevalent in individuals who were diagnosed with tuberculosis or had an unknown route of HIV infection, while was more common in people who used Medicare/self-paid medications. The rapid ART group had an LTFU rate of 3.3%, as opposed to 5.0% in the delayed initiation group. Moreover, the rapid ART group had a much reduced virological failure rate (0.6% *vs*. 1.8%). Rapid ART individuals had a reduced likelihood of LTFU (adjusted hazard ratio: 0.65, 95% CI: 0.44 - 0.96) and virological failure (adjusted odd ratio: 0.35, 95% CI: 0.12 - 0.80).

**Conclusion:**

Under China's Treat-All policy, rapid ART initiation reduced the risk of LTFU and virological failure. The real-world data indicated that rapid ART initiation is practicable and beneficial for Chinese people with HIV, providing evidence and guide for its widespread implementation and scaling up.

**Disclosures:**

**All Authors**: No reported disclosures

